# From top to bottom: Cell polarity in Hedgehog and Wnt trafficking

**DOI:** 10.1186/s12915-018-0511-x

**Published:** 2018-04-10

**Authors:** Ana-Citlali Gradilla, David Sanchez-Hernandez, Lucy Brunt, Steffen Scholpp

**Affiliations:** 1 Centro de Biología Molecular ‘Severo Ochoa’ (CSIC-UAM), Universidad Autónoma de Madrid, Nicolás Cabrera 1, Cantoblanco, E-28049 Madrid, Spain; 2 Living Systems Institute, School of Biosciences, College of Life and Environmental Sciences, University of Exeter, Exeter, EX4 4QD UK

## Abstract

Spatial organization of membrane domains within cells and cells within tissues is key to the development of organisms and the maintenance of adult tissue. Cell polarization is crucial for correct cell–cell signalling, which, in turn, promotes cell differentiation and tissue patterning. However, the mechanisms linking internal cell polarity to intercellular signalling are just beginning to be unravelled. The Hedgehog (Hh) and Wnt pathways are major directors of development and their malfunction can cause severe disorders like cancer. Here we discuss parallel advances into understanding the mechanism of Hedgehog and Wnt signal dissemination and reception. We hypothesize that cell polarization of the signal-sending and signal-receiving cells is crucial for proper signal spreading and activation of the pathway and, thus, fundamental for development of multicellular organisms.

## Hedgehog and Wnt—two major signalling networks

The signalling proteins of the Hedgehog (Hh) and Wnt families are both major organizers of development, directing cell differentiation and tissue patterning. In adult life they remain essential for maintaining tissue homeostasis. Both Hh and Wnt molecules are fundamental to many vital processes such as cell proliferation, cell migration, cell differentiation and axonal path finding. These fundamental processes contribute to embryonic development as well as playing a role in disease processes like tumour genesis [1–3]. Remarkably, although Hh and Wnt are two distinct signalling pathways, with different ligands, receptors, effectors and targets, new research is revealing striking parallels regarding their mechanisms for signal production, distribution, release and reception. Production of these signalling molecules is confined to a cell cluster, and the signal is then transported over considerable distance to organize the neighbouring tissue. Furthermore, both molecules also share characteristic post-translational processing with the addition of lipids, which results in anchoring to the plasma membrane. Strong membrane association of the signalling factors impedes their free diffusion towards receiving cells, and thus, alternative transport modes have been investigated.

Two membrane-bound mechanisms are proposed: transport through extracellular vesicles and/or signalling filopodia called cytonemes [4]. Besides their emerging role in cancer biology and diagnostics, extracellular vesicles play a specific role in cell-to-cell communication [5, 6]. Exovesicles have been found to carry Hh and Wnt proteins and activate the corresponding signalling pathways in cells they fuse or interact with. In addition, we and others have experimentally analysed cytoneme-based signal transport as a mechanism that facilitates the distribution of the membrane anchored Hh and Wnt ligands towards reception [7–13]. Signalling through filopodia has also been described for other signalling pathways, such as the EGF, FGF, Notch and Bmp pathways, suggesting that this might be a general mechanism used by cells and thus cellular regulation processes could be similarly shared [4]. In addition to exosomes and cytonemes, further intercellular trafficking of signalling molecules has been reported. These include signalling dissemination through processed diffusible ligands, soluble ligand multimers, special carrier proteins, and lipoproteins; however, these are the focus of other reviews [14–16].

The cell membrane is far from homogeneous and evidence of differential compartmentalization of membranes for cell function is constantly being revealed. Epithelial cell polarization includes the specification of an apical domain, towards the luminal side of a tissue, and a basal membrane, in contact with the extracellular matrix, while polarized mesenchymal cells distinguish a front and rear axis. This not only distinguishes two sides of the cell in the planar dimension, but also provides distinct membrane properties and components. Such a spatial organization of the cell is known to be essential for morphogenetic processes during development as well as to maintain tissue homeostasis. For example, changes in polarity are related to an increase of proliferation in tumour development [17]. However, the mechanisms behind this process are just starting to be unveiled. The secretion of exovesicles, filopodia formation and intracellular trafficking are strongly linked to cell polarity regulation; thus, we hypothesize that the study of the cells own spatial organization is significant to our understanding of signalling processes in complex tissues.

Latest advances in intracellular signal trafficking, exovesicle generation and cytoneme-mediated signal transport show remarkable similarities between the Hh and Wnt pathways. These processes are tightly linked to the spatial cellular organization of the signal-producing cell. In addition the signal-receiving cell is also polarized to ensure proper signal reception and activation of the signalling cascades. In this review, we examine this research from a cell polarity perspective, stressing common and unique features, exposing a general mechanism for lipid-modified ligands and examining its links to cell polarity regulation.

## Hh and Wnt molecules are both lipid-modified and strongly associated with membranes

**Fig. 1.**
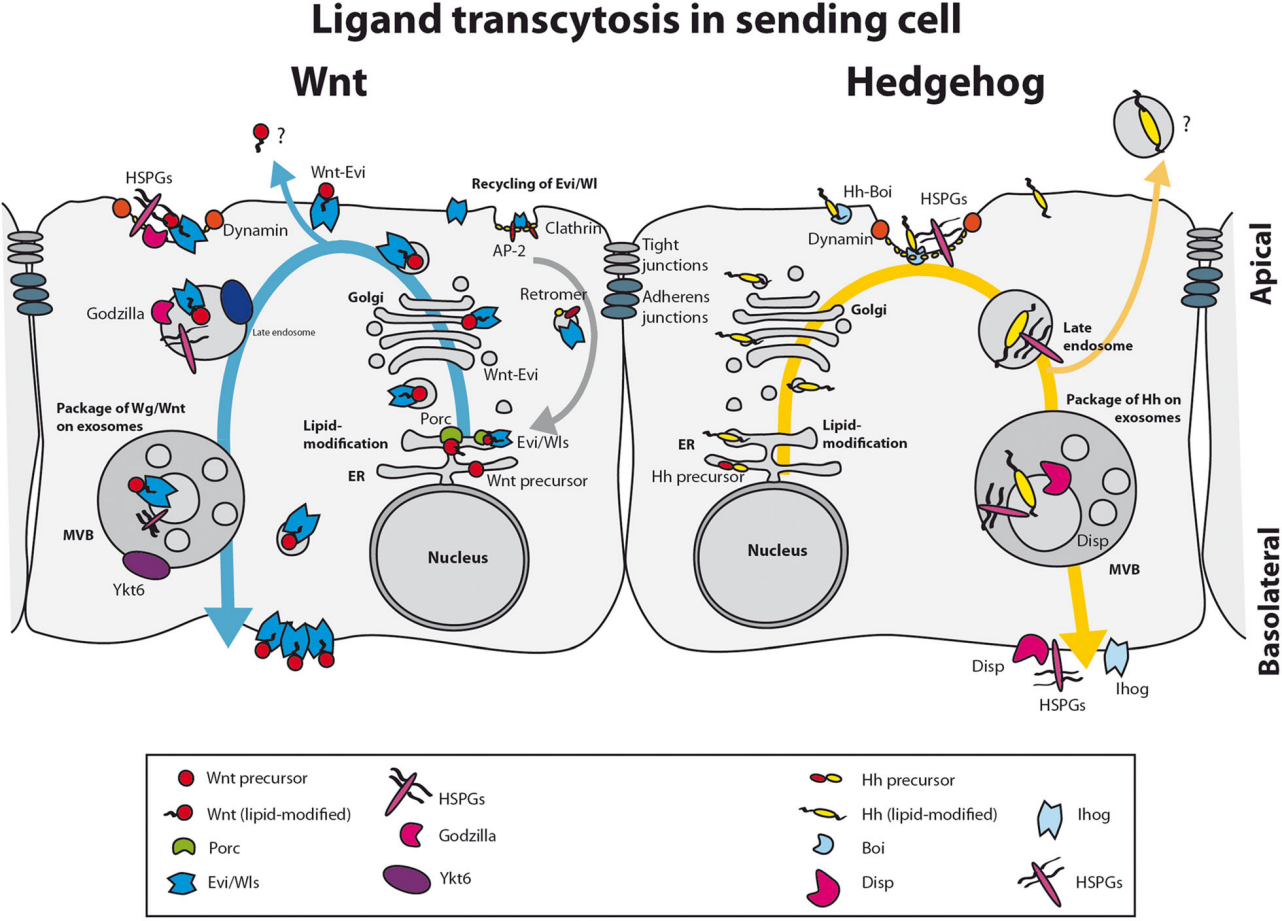
Apical ligand recycling in source cell. Endocytosis-regulated Wnt/Wg and Hh secretion. After formation and lipid modification of the ligand, chaperones transport the ligands to the apical side of the source cell. The ligands get re-endocytosed and packaged on exosomes for subsequent secretion at the basolateral side

Hh and Wnt protein family members are produced as non-functional precursors (Fig. 1). Both molecules are subject to different post-translational modifications, essential for their final activity, including secretion and signalling. Despite the differences in their synthesis and processing, the result is similar: highly lipid-modified signalling molecules that are strongly attached to cellular membranes.

In the case of Wnt/Wg proteins two kinds of modifications have been described, palmitoleic acid addition and N-glycosylation, both taking place in the endoplasmic reticulum (ER). The ER multipass O-acyltransferase Porcupine (Porc) directs palmitoylation and maturation of Wnt proteins [18–20]. Lipid modifications are essential for correct secretion and signalling [18, 21, 22]. Mutations in the cysteine residues subject to palmitoylation result in a significant loss of activity and redistribution of Wnt proteins in both vertebrates and *Drosophila* [18, 23, 24]. Recent data show that cleavage of the essential palmitoleate moiety by the carboxylesterase Notum is an important regulatory process to reduce Wnt signalling range [25]. Extracellular levels of Notum in the blood serves as a biomarker for Wnt-driven cancer [26]. In the case of glycosylation, the number of glycosylation residues varies between the different Wnts and it is possible this modification is dispensable for its activity [27, 28].

Hh is initially synthesized as a 45-kDa precursor before undergoing autocatalytic cleavage in the ER [29, 30]. The cleaved C-terminal region (Hh-C) is degraded via the proteasome [31] while the N-terminal part (Hh-N) containing the signal activity is dually lipidated [32]. First, an autocatalytic cholesterol addition in the C-terminus is made [31, 33], followed by the palmitoylation of an N-terminal conserved cysteine [34–36]. Hh-N lacking cholesterol or palmitic acid attachment is able to signal but not to fully activate targets in responding cells [37–40]. Therefore, parallel to the case of Wnt, these lipid modifications are required for the complete activity of the protein. In addition, the indispensable molecules for Hh signalling, Dispatched (Disp), which is required for the release of Hh from producing cells, [41–45] and the soluble extracellular matrix (ECM) factor Scube2 [46–50] require the correct lipidation of Hh to function. Loss-of-function mutants for Disp show accumulation of trapped lipid-modified Hh in unresponsive producing cells, but not unmodified Hh [41, 42, 45], showing that lipid modifications are essential for the required Hh dispersion. In vertebrates, Scube2 is required for processing and bioactivation of Sonic Hedgehog (Shh), one of three mammalian Hh homologues [51]. Scube2 is not present in flies, but the diffusible protein Shf/Wif-1 is also similarly indispensable for lipid-modified Hh long-range signalling in the wing disc [52–55].

As described above, activity of Hh and Wnt proteins is dependent on correct lipid modification. On this same line of thought, cholesterol addition to Shh has also been shown to enhance long-range signalling [56]. Besides a potential signal function, the immediate consequence of lipidation is the anchoring of the molecule to cell membranes, which would restrict free diffusion but, paradoxically, could be in favour of long-distance distribution. In agreement with this observation, two membrane-dependent mechanisms for Hh/Wnt distribution have been proposed: movement by means of membranous organelles such as cytonemes and through exovesicles [9, 11, 16, 57]. Indeed, a membrane-tethered form of Wg replacing the endogenous one in *Drosophila* has been reported as able to signal and produce patterning. The authors suggest dispersion is dispensable and instead propose that all cells initially express the ligand and, by dynamic decline of expression, patterning is achieved. [58]. However, the possibility of additional direct membrane–membrane distribution mechanisms at a distance such as through cytonemes has not been ruled out. Thus, lipid modification of Wnt/Hh ligands is a prerequisite for signalling and membrane anchoring, which we propose allows their inclusion in the distribution mechanism that includes pathway-crucial elements for proper signalling.

## Hh and Wnt molecules undergo apico-basal recycling prior to their distribution

After processing, ligands reach the cell membrane (Fig. 1). Remarkably, both Hh and Wnt molecules require similar complex and precise intracellular trafficking in the producing cells before their release [43, 53, 59, 60]. The Golgi transmembrane protein Evi/Wls plays a crucial role in the trafficking of mature Wnts to the cell membrane [61–63]. Evi/Wls and Wnt proteins are transported together to the cell surface before being re-endocytosed, and in Evi/Wls loss of function experiments Wnt appears trapped in the secretory pathway, blocking its final release. Interestingly, Evi/Wls is then recycled and retrogradly transported from plasma membrane back to the ER [64]. This process requires the retrograde endosomal protein-sorting machinery, the retromer complex, as Evi/Wl is degraded in the lysosome in retromer mutants, resulting in reduction of Wnt secretion [65–69]. Thus, after membrane presentation Wnt is re-endocytosed in a Dynamin-dependent process before its final secretion [70, 71]. Cellular compartmentalization of this procedure might be a key regulatory aspect of signalling.

In this line of thought, recycling of *Drosophila* Wg from the apical surface is regulated by the ubiquitin ligase RNF Godzilla that targets the Snare-complex protein Synaptobrevin [60]. In this process, Wg protein is routed from early apical endosomes to the basolateral surface where signal secretion takes place. Previous research also points out an additional role for glycosylation in the polarized sorting of Wnt3a for basolateral secretion and in apical secretion of Wnt11 [72]. Different types of glycosylation target different Wnts for their respective sorting in the epithelial polarized cells, where for example the crucial Evi/Wls localizes basolaterally and is necessary for Wnt3a basal secretion but not for Wnt11 apical secretion [72]. Thus, prior to secretion, Wnt protein follows a highly regulated intracellular recycling route in the ligand-producing cell.

Analogous to the Wnt protein, Hh in the source cells also needs fine regulation of its intracellular traffic before distribution. Besides disagreements regarding concentration gradient contribution, two secreted Hh populations have been described in *Drosophila* epithelia*,* an apical and a basolateral one [11, 43, 53, 59, 73, 74]. Here again as for Wnt/Wg, dynamin-dependent endocytosis is required for proper Hh signalling activity. Dynamin mutants in the producing cells of the *Drosophila* wing disc accumulate Hh in the apical domain [43, 73, 75], while interfering with Rab proteins (e.g. Rab5, Rab4 and Rab8), implicated in the first recycling step, results in Hh subapical accumulation and a reduction in signalling [43, 59]. Two different routes for the secreted populations have been proposed: in the first one we argue that Hh is endocytosed to then reach the basolateral membrane for long range signalling via cytonemes and exovesicles [11, 43, 75]; while in a second one, Hh endocytosis from the apical membrane allows its inclusion within exovesicles to be finally distributed for distant signalling through the apical membrane again [59].

Supporting a mainly basolateral distribution of Hh, the crucial Disp protein is basolaterally localized in polarized epithelium [43, 76, 77]. In addition, the cell surface heparan sulfate proteoglycans (HSPGs) Dally-like (Dlp) and Dally, essential for Hh signalling activity, are also proposed to be involved in Hh recycling to the basolateral membrane [43]. Dally and the Hh co-receptor Brother of *Ihog* (Boi) are suggested as key factors in the retention of apical Hh for subsequent re-internalization and basolateral redirection [53]. In a different interpretation Dally has been suggested to facilitate long-range signalling through the apical membrane, after the cleavage of its GPI anchor by the hydrolase Notum [78]. However, additional experiments have suggested that Notum is not required for Hh release and does not function in the cleavage of the Dally GPI anchor but rather limits Wg/Wnt signalling by cleavage of the palmitoleate adduct from Wnt proteins [25]. On the other hand, Dlp co-localizes and interacts with Disp at the basolateral membrane [43], where the Hh co-receptor Ihog also localizes and holds Hh for its distribution to receiving cells [53]. Furthermore, in agreement with Hh recycling to basolateral membrane, recent vertebrate experimental data revealed a defect in apico-basal distribution of Shh, with less basolateral morphogen on Shh producer cells, when deficient for the heparin sulfate synthase Ext1, during lung development [79]. Therefore, we conclude that polarized localisation of Hh-related proteins such as Ihog and the involvement of HSPGs within intercellular trafficking are key for Hh redistribution.

Furthermore, intracellular trafficking and cell polarity could be interdependent [80]. The endocytosis process could be directing an endosomal sorting target, different from degradation, as well as partly maintaining a polarized status of the membrane. Intracellular trafficking regulation through the endosomal sorting ESCRT complex is indeed involved in cell polarity definition, both in apico-basal epithelial polarity as well as front-rear polarity in migrating cells [81]. In this context, several members of the ESCRT complex have been shown to be involved in both Hh and Wnt signalling, probably through their role in polarized endosomal sorting as well as exovesicle formation. Further evidence of endosomal sorting being required for signalling has recently been shown through the Arf6/Hh interaction, which impedes Hh endosome targeting to lysosomal degradation, thus allowing its traffic to be targeted to signal competent endosomes [82].

Endocytosis and intracellular endosomal sorting are crucial processes prior to ligand secretion in both the Hh and Wnt/Wg source cells. We hypothesize that polarity of the cell membrane in ligand-producer cells is important to the signalling process in general. In support of this, apico-basal trafficking has also been proposed for the lipid modified EGF ligand Spitz [83]. However, all evidence to date refers to epithelial apico-basal polarity and whether a different compartmentalization or polarity arrangement of the cell membrane could be important for signalling between mesenchymal cells is a major outstanding question.

In summary, the polarization of the source cell determines the route of secretion of the ligands of the Hh and Wnt family. This has an important influence on the mode of transport, the signalling range and the reception of the ligand, which we will discuss in the following sections.

## Exosomes and cytonemes disseminate Hh and Wnt proteins

**Fig. 2.**
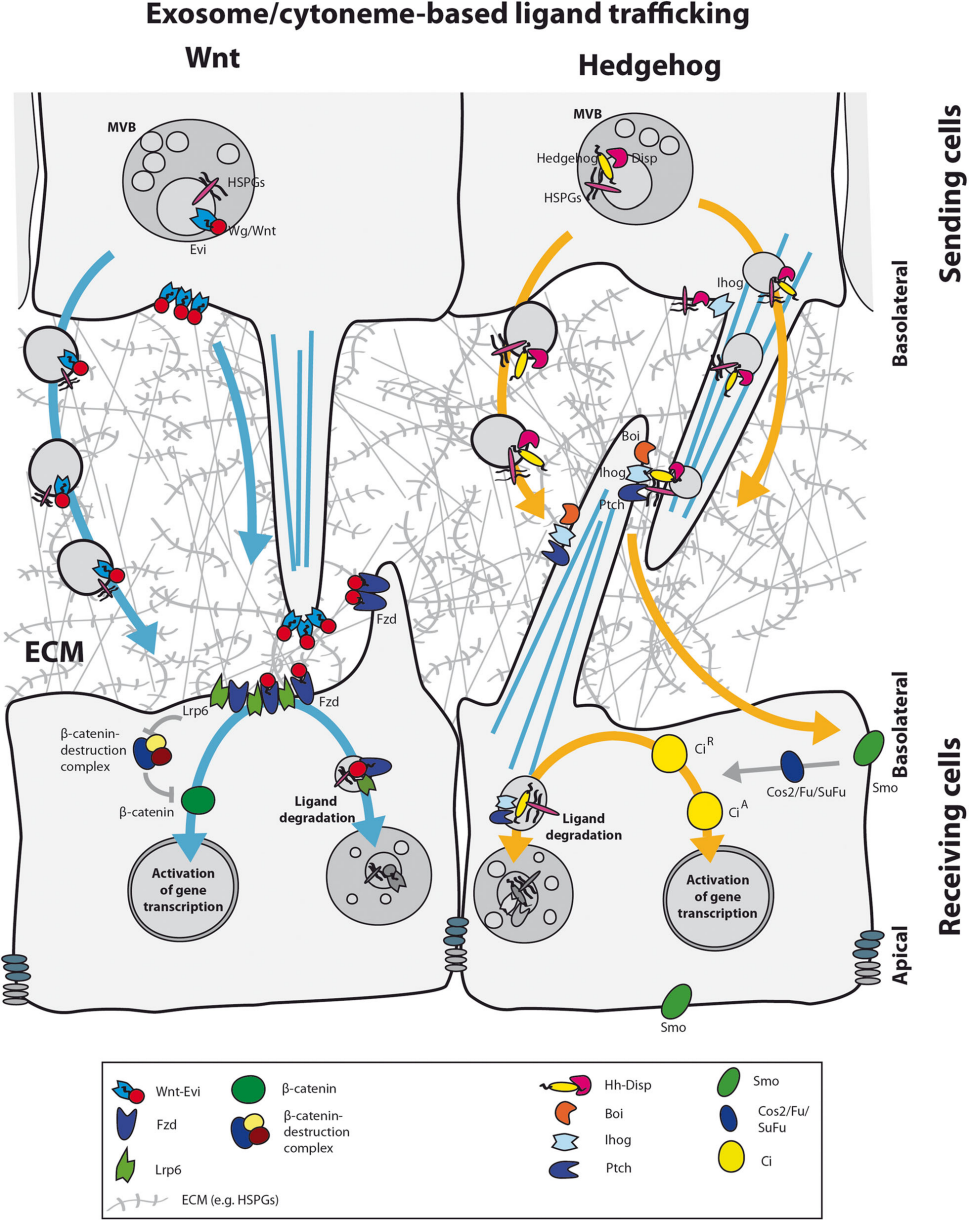
Ligand trafficking at the basolateral side. Wnt/Wg and Hh traffic from the basolateral side of the source cell to the receiving cell on exosomes or cytonemes. At the basolateral side of the receiving cell, the ligands bind to the receptors and co-receptors, respectively. Consequently, signal pathway activation and ligand–receptor degradation take place in the target cell

In the last few years, experimental data show that exovesicles (EVs) could be a key factor in the secretion of the hydrophobic molecules Wnt and Hh (Fig. 2) [6, 11, 57, 74, 84, 85]. This mechanism requires the participation of multivesicular bodies (MVBs), where the cargo is loaded from endosomes prior to exocytosis, and is dependent on ESCRT and Snare family members [86]. Indeed, components of the ESCRT machinery are strongly implicated in Wnt and Hh distribution and signalling function, reported both in cell culture as well as in vivo experiments [6, 11, 74, 87]. Supporting this model, the molecules Hh and Disp have been shown to be present, facing the extracellular side, on the membrane of exosomes [11]. In *Drosophila*, the Wg and Evi/Wls complex has also been observed within exosomes [84] in a process requiring the exocytic G-protein Rab11 and Myosin 5A [88].

Strongly challenging free diffusion models, experimental evidence for cytoneme-mediated signalling has significantly increased in the last 10 years, coinciding with the great advance in imaging techniques. Cytonemes are actin-based filopodia functionally specialized in signalling [4, 89]. For both Wnt and Hh, we and others have identified cytonemes directly influencing signalling, as short-term interruption of the cytoskeleton machinery for filopodial establishment in source cells results in shortening of cytonemes and the signalling range [7, 12–14]. Signalling through these types of protrusions has been reported in *Drosophila* as well as vertebrate systems, mediating the transport of molecules such as Notch [90–93], EGF [94, 95], FGF [95], BMP [96, 97], Wnt [7, 93, 98] and Hh [8–10, 12, 13]. Thus, cytonemes as an intercellular communication structure are likely to have a pivotal role in morphogenesis during development as well as in adult tissue maintenance. It therefore follows that filopodia regulation mechanisms are highly relevant to understanding signalling function. Understanding cytoneme establishment and cargo upload are now critical aspects to be determined. In turn, filopodia formation and cargo upload might be directly linked to cell polarity regulation, clearly implicating compartmentalization of cell procedures [99].

The cytoneme and exovesicle models should not be seen as two independent routes for dissemination. Exovesicles containing Wnt/Hh are not sufficient for the complete activation of target genes in vitro, suggesting that an extra mechanism for signalling via vesicles is required to accomplish signalling [11, 57, 85]. We and others have shown that cytonemes are required for Hh restricted long distance signalling [9, 12, 13] and exovesicles containing Hh are proposed to move along cytonemes to reach receiving cells [11]; thus, signalling through exovesicles might need to be mediated by direct cell–cell contact to activate signalling completely, which would not occur via exovesicle transport alone. In addition, Wg exosomes have been shown to travel along axons and be released into the synaptic cleft of neuromuscular junctions in *Drosophila*; thus, a similar mechanism may be at play in cytonemes. Wg exosomes then activate the Wnt signalling cascade of the post-synaptic neuron [84]. Furthermore, endocytosis has been shown to be necessary for cytoneme-mediated signalling [97] and thus it is likely to require specific endosomal sorting towards the localized cytoneme. Wnt signalling is activated after endocytosis of the Wnt–receptor complex [100].

However, a limitation of the combined membrane-bound transport mechanisms would be to grasp the means by which soluble factors such as Scube 2 and Shifted (in *Drosophila)* influence signal spreading [51, 53], as well as how factors with an inhibitory effect over the pathways such as the Hedgehog Interacting Protein (Hip) [101] and secreted Frizzled Related Proteins (sFRPs) exert their effects [102]. To date whether the signalling molecules travelling on cytonemes are localized inside or outside the filopodia remains unclear. Thus, one possibility is that either through travelling or at the release point, the membrane-anchored signalling molecules are exposed to the extracellular space and interacting with such soluble factors. Reinforcing this possibility, both the Hh and Wnt molecules are located at the outside of EVs [11, 57].

In the section above, we have described that cytonemes and EVs are modes to spread lipid-modified ligands such as Hh and Wnt in a tissue. The formation of these carriers requires a machinery of proteins routing the ligand from the ER/Golgi through the endocytic pathway to the locations at the cell membrane where these transport carriers can be formed.

## Transport of signalling molecules in a polarized cell

Basolateral polarization of filopodia formation against the apical establishment of micro-villi, and lateral lamelipodia-like protrusions, has been shown for epithelial cells [103]. This apico-basal graded distribution of protrusions is also regulated by an apico-basal concentration gradient of the Rho GTPase Rac, which is in turn defined by polarity proteins, possibly through direct inhibition of Rac activity on the apical side of the cell [99, 103]. Experimental intervention using dominant-negative Rac in the *Drosophila* notum primordium affected Delta-Notch signalling through basal filopodia, essential for establishment of the highly organized bristle pattern [90]. These filopodia are basally localized and dynamic, and signal through transient direct filopodia–filopodia contact, establishing, as a result, intermittent cell–cell signalling for gradual refinement of pattern during lateral inhibition [90]. Hh signalling cytonemes in the *Drosophila* wing disc epithelium also mainly extend basally. Emanating from both the ligand source cells as well as receiving cells, they form and break contacts dynamically, with contact activating signalling [9, 12, 13]. In addition, our electron microscopy analysis of the wing disc revealed the presence of microvilli structures only on the apical surface of the epithelium [11], in concordance with the polarized organization of protrusion establishment previously described for notum epithelia [103]. Recycling of Hh from the apical to the basal membrane as a previous step for its distribution is also in accordance with the polarization of the signalling filopodia.

A remarkably similar recycling process has been reported for the *Drosophila* Wg [60], again linking polarization to the mechanism for dispersion (Fig. 1). During zebrafish development we have described cytonemes to carry Wnt8a at their tips, transporting the ligand from source cells towards receiving cells until direct contact is reached [7]. At the source cell, Wnt on cytoneme tips initiates the formation of the ligand–receptor complex—the so-called Wnt signalosome—which is a prerequisite for signal activation. Interestingly, in the case of the source cell the ligand Wnt8a clusters at the plasma membrane where it can recruit the transducer of CDC42-dependent assembly protein 1 (Toca-1) and consecutively activate a filopodia nucleation complex for localized filopodia initiation [7, 104]. Hence, intracellular trafficking of the Wnt ligand could also be key to the spatial localization of protrusion establishment in vertebrates and is likely to be linked to cell membrane polarization.

Other factors recently found to direct basolaterally localized cell membrane protrusions are the G-protein-coupled receptors and stem cell markers Lgr5 and Lgr4. Lgr4/5 activation by R-spondin ligands potentiates Wnt/β-catenin signalling. Interestingly, their presence at the plasma membrane after blocking their internalization robustly promotes filopodia, and these protrusions are capable of performing signalling molecule conveyance and share defined characteristics of cytonemes such as being long fragile actin-rich filopodia (up to 100 μm) [105]. Consistent with this, recent findings suggest that Lgr5 primarily functions via the Rac1 pathway to enhance actin polymerisation and strengthen cell–cell adhesion in stem cells in the intestinal crypt and colon cancer cells [106]. In *Drosophila* there is evidence of long distance recruitment of the neuropeptide Bursicon by the ortholog Lgr2, although the mechanisms remain unknown [107].

These findings suggest a potential role for the receptors, comprising the hardware for potential long-distance signalling in stem cells, but also adds to the hypothesis of a generally shared mechanism for distant cell–cell communication through basal filopodia. Not only could cell intrinsic cues favour the formation of filopodia at the basal side, but also external signals could locally facilitate the emergence of cytonemes.

## Polarized formation of cytonemes in the receiving cell

Apico-basal polarity in signal reception is another important on-going research question (Fig. 2). It is likely to have important implications for our understanding of signalling regulation. In vertebrates the requirement of cilia, an apical cytoplasmic microtubule-based extension, for Hh signalling (i.e. Shh, Ihh) has been clearly demonstrated [108–110]. These data along with the finding of Shh/Ptch1 co-localization within cilia, has led to the possible misconception that cilia are the cellular compartment for Hh reception [111]. Interestingly, interaction between ligand-carrying cytonemes and receiving cell cytonemes containing the co-receptors Cdo/Boc has been observed during chicken limb bud development, suggesting a cilia-independent Hh reception process through cytoneme-mediated signalling [10].

In some cell types transduction of Hh signal after signal reception is independent of a cilia-like structure. In *Drosophila*, although most cells lack cilia, compartmentalization of Hh signal transduction might still occur within the plasma membrane due to Smoothened activation mechanisms [112, 113], while cytoneme-mediated Hh reception takes place at the basal side of receiving cells [12, 13]. In the wing disc epithelium, the Hh receptor Ptc localizes on basolateral cytonemes emanating from Hh responding cells; and experiments blocking dynamin-dependent rapid endocytosis of the receptor complex show the clear basal localization of extracellular Hh/Ptc complex [13]. Moreover, basal contact sites of producer and receiving cell cytonemes was revealed by high-resolution imaging in the *Drosophila* wing imaginal disc. In the GRASP technique (GFP Reconstitution Across Synaptic Partners), complementary fragments of a fluorescent molecule are expressed in each distinct cellular compartment. The GRASP technique showed cytoneme-to-cytoneme contact all along the receiving territory and importantly co-localization of both the receptor Ptc and the ligand Hh was shown at contact sites [13]. Basal cytoneme-mediated Hh uptake was also observed after monitoring imaging of fluorescent Hh, Ptc and Smo at physiological levels, providing further data of intracellular Hh distribution at source cells [12] and in favour of an apico-basal polarized signalling process. In addition, despite the unknown mechanisms for actual molecule release and signal activation at the contact and reception site, either from cytonemes or EVs, there is strong parallel evidence for the requirement of a proteolytic shedding process carried by extracellular matrix metalloproteases together with Scube2 and HSPGS [114]. Thus, basolateral cytonemes could closely approximate the area where final membrane shedding and Hh release occur for signal activation (further reviewed in [114]).

In a similar signalling scenario where vertebrate polarized cells of the developing neural tube display a Shh concentration graded response, Shh uptake might also occur basally. In this context, although two externalized Hh pools are found, most extracellular Hh is localized on the basal side of the ventral neural tube, which is also the closest side to the Hh source, the notochord [111]. Furthermore, in the retina neuro-epithelium Shh and the co-receptor Cdo co-localize at the basolateral side, where also filopodia-like structures are stabilized by Cdo [115]. Basal signal reception then is in accordance with the described required recycling of the Hh ligand.

A parallel mechanism has been shown for Wg/Wnt proteins [60]. Basal dynamic cytonemes have been shown to transport the Wnt receptor Frizzled (Fzd) from myoblast progenitors of flight muscles towards Wg source cells in the wing disc epithelium, forming a Wg/Fzd complex that then moves in a retrograde direction [93]. Likewise, the chicken Wnt receptor Fzd7 has been observed on filopodia emanating from dermomyotome cells [116]. In addition, in *Xenopus* the Wnt co-receptor Lrp6 is asymmetrically localized to the basolateral membrane in ectodermal blastomeres [117]. This, together with the mainly basal distribution of Wg across the receiving territory in the *Drosophila* wing disc, might indicate similar reception mechanisms for long-distance signalling.

We have discussed the importance of cell polarization in signal dissemination and, in addition, the increasing evidence that the target cell is similarly polarized. This leads to the question: how are receiving cytonemes influenced by polarization? Based on the presented work, we suggest that cell polarity in the target cell influences the formation of responding cytonemes but also the localization of the receptor complex and signal transduction.

## Basal formation of cytonemes and the extracellular matrix

As well as via cell intrinsic cues, basal polarization of filopodia might also be linked to the interaction of the basal membrane with the extracellular matrix (ECM), which provides an adhesive framework for the filopodia (Fig. 2). In this context, we have shown that *Drosophila* basal Hh cytonemes are unable to extend across large *ttv* mutant clones where HSPG assembly in the ECM has been impaired [9]. Similarly, FGF and BMP cytoneme extension is experimentally hampered by the reduction of HSPGs and laminin within the ECM, on which cytonemes would normally grow during dorsal air sac development [118]. Interestingly, in the latter case the planar cell polarity (PCP) regulators Prickle (Pk) and Van Gogh (Vang) are reported as responsible for maintaining the normal extracellular levels of the HSPGs Dally and Dally-like (Dlp) [118]; however, details of the regulatory mechanism are still unknown and could be unrelated to the known role of Pk and Vang in the definition of tissue planar polarity.

The requirement of HSPGs for cytoneme spread has again been demonstrated for Hh signalling in the wing disc epithelium where the signalling filopodia extend aberrantly through Dally and Dlp mutant territory [13]. Moreover, receiving-cell cytonemes over-expressing Dally or Dlp acquire stability when interacting with cytonemes over-expressing the co-receptor Ihog and emanating from Hh producer cells, indicating a potential role for HSPG/Ihog interaction in *trans* during cytoneme growth [13]. Additionally, a recent publication describes a novel role for the Hh co-receptors Ihog/Boi in cell adhesion, where they are crucial for correct segregation of posterior and anterior cell populations [119]. Ihog retains Hh on the cell surface mainly at the basolateral side of the *Drosophila* wing disc for distribution, presentation and subsequent signal reception at cytoneme contacts [13]. Ihog then would allow internalization of Hh receptor Patched (Ptc)/Hh complexes. It is this Ptc/Hh endocytosis that directs Ihog to degradation in receiving cells, resulting in a protein spatial pattern that is the reverse of Ptc expression, with less Ihog on the Hh signalling region [119]. These decreased Ihog levels seem to reduce cell–cell adhesion [119] and, interestingly, this coincides with the locations where Hh cytoneme extension would take place [9, 13]. In summary, the extracellular matrix provides the substrate for cytonemes to extend. We speculate that the interaction of cell membranes with the ECM may have a role in induction, but also in stabilisation and direction, of these fragile structures.

However, regulation of cytoneme orientation is still a key outstanding question and might result from the combination of several PCP regulators, interaction with the ECM, and cytoneme dynamics regulation in *trans*. Evidence of the PCP pathway influencing cytoneme appearance is scarce and further research is needed, as so far it is unclear if the PCP pathway is activated in the source and/or in the receiving cells and how its activity in cytoskeletal remodelling could connect to cytoneme formation.

## Concluding remarks and open questions

Lipid modifications are key features of the signalling molecules Hh and Wnt. These modifications can influence apico-basal localisation [43, 76] and this in turn is likely to define the secretion mechanism of these two proteins. We compared recent data regarding Hh and Wnt secretion, distribution and reception, revealing a potential general model for lipid-modified ligands. We provide evidence that trafficking in the ligand-producing cells shifts membrane-anchored molecules from apical membrane to basal membrane, targeting distribution through cytonemes. We hypothesize that apical ligand re-endocytosis is required for acquiring membrane identity and inclusion into a membrane-bound dispersion mechanism such as exovesicles and/or cytonemes at the basolateral side. Furthermore, signal reception might also be polarized; recent evidence in Hh and Wnt signalling suggests a basal localization of both receiving cytonemes and reception complexes. These conclusions are in line with the possible coordination of vesicle trafficking and protrusion formation with regard to the cytoskeleton and membrane spatial arrangement of cells.

Critical areas regarding a cytoneme-mediated mechanism for signalling still need to be explored, including their establishment and cargo uploading, and it is likely these steps also involve cell polarity regulation elements. Understanding the polarized behaviour of vesicle genesis and transport, as well as cellular protrusions for signalling, is thus a vast area for future research, one that is increasing in interest as emerging research reveals their role in a greater variety of signalling scenarios, both during development and in adult tissue maintenance. Identification of hub points of signal trafficking and cellular polarity might allow their identification as potential targets in drug discovery and add to the achievement of greater insight into coordinated developmental processes.

## References

[1] Nusse R, Clevers H (2017). Wnt/beta-catenin signaling, disease, and emerging therapeutic modalities. Cell.

[2] Briscoe J, Therond PP (2013). The mechanisms of Hedgehog signalling and its roles in development and disease. Nat Rev Mol Cell Biol.

[3] Simon E, Aguirre-Tamaral A, Aguilar G, Guerrero I. Perspectives on intra- and intercellular trafficking of Hedgehog for tissue patterning. J Dev Biol. 2016;4:34. 10.3390/jdb4040034 PMC5831803 29615597

[4] Kornberg TB, Roy S (2014). Cytonemes as specialized signaling filopodia. Development.

[5] Hessvik NP, Llorente A (2017). Current knowledge on exosome biogenesis and release. Cell Mol Life Sci.

[6] Parchure A, Vyas N, Ferguson C, Parton RG, Mayor S (2015). Oligomerization and endocytosis of Hedgehog is necessary for its efficient exovesicular secretion. Mol Biol Cell.

[7] Stanganello E, Hagemann AI, Mattes B, Sinner C, Meyen D, Weber S (2015). Filopodia-based Wnt transport during vertebrate tissue patterning. Nat Commun.

[8] Rojas-Rios P, Guerrero I, Gonzalez-Reyes A (2012). Cytoneme-mediated delivery of hedgehog regulates the expression of bone morphogenetic proteins to maintain germline stem cells in Drosophila. PLoS Biol.

[9] Bischoff M, Gradilla AC, Seijo I, Andres G, Rodriguez-Navas C, Gonzalez-Mendez L (2013). Cytonemes are required for the establishment of a normal Hedgehog morphogen gradient in Drosophila epithelia. Nat Cell Biol.

[10] Sanders TA, Llagostera E, Barna M (2013). Specialized filopodia direct long-range transport of SHH during vertebrate tissue patterning. Nature.

[11] Gradilla AC, Gonzalez E, Seijo I, Andres G, Bischoff M, Gonzalez-Mendez L (2014). Exosomes as Hedgehog carriers in cytoneme-mediated transport and secretion. Nat Commun.

[12] Chen W, Huang H, Hatori R, Kornberg TB (2017). Essential basal cytonemes take up Hedgehog in the Drosophila wing imaginal disc. Development.

[13] Gonzalez-Mendez L, Seijo-Barandiaran I, Guerrero I. Cytoneme-mediated cell-cell contacts for Hedgehog reception. elife. 2017;6:e24045. 10.7554/eLife.24045 PMC5565369 28825565

[14] Port F, Basler K (2010). Wnt trafficking: new insights into Wnt maturation, secretion and spreading. Traffic.

[15] Muller P, Schier AF (2011). Extracellular movement of signaling molecules. Dev Cell.

[16] Stanganello E, Scholpp S (2016). Role of cytonemes in Wnt transport. J Cell Sci.

[17] Elsum I, Yates L, Humbert PO, Richardson HE (2012). The Scribble-Dlg-Lgl polarity module in development and cancer: from flies to man. Essays Biochem.

[18] Willert K, Brown JD, Danenberg E, Duncan AW, Weissman IL, Reya T (2003). Wnt proteins are lipid-modified and can act as stem cell growth factors. Nature.

[19] Takada R, Satomi Y, Kurata T, Ueno N, Norioka S, Kondoh H (2006). Monounsaturated fatty acid modification of Wnt protein: its role in Wnt secretion. Dev Cell.

[20] Rios-Esteves J, Resh MD (2013). Stearoyl CoA desaturase is required to produce active, lipid-modified Wnt proteins. Cell Rep.

[21] Janda CY, Waghray D, Levin AM, Thomas C, Garcia KC (2012). Structural basis of Wnt recognition by Frizzled. Science.

[22] Kumar S, Zigman M, Patel TR, Trageser B, Gross JC, Rahm K (2014). Molecular dissection of Wnt3a-Frizzled8 interaction reveals essential and modulatory determinants of Wnt signaling activity. BMC Biol.

[23] Couso JP, Martinez AA (1994). Notch is required for wingless signaling in the epidermis of Drosophila. Cell.

[24] Nusse R (2003). Wnts and Hedgehogs: lipid-modified proteins and similarities in signaling mechanisms at the cell surface. Development.

[25] Kakugawa S, Langton PF, Zebisch M, Howell S, Chang TH, Liu Y (2015). Notum deacylates Wnt proteins to suppress signalling activity. Nature.

[26] Madan B, Ke Z, Lei ZD, Oliver FA, Oshima M, Lee MA (2016). NOTUM is a potential pharmacodynamic biomarker of Wnt pathway inhibition. Oncotarget.

[27] Komekado H, Yamamoto H, Chiba T, Kikuchi A (2007). Glycosylation and palmitoylation of Wnt-3a are coupled to produce an active form of Wnt-3a. Genes Cells.

[28] Mason JO, Kitajewski J, Varmus HE (1992). Mutational analysis of mouse Wnt-1 identifies two temperature-sensitive alleles and attributes of Wnt-1 protein essential for transformation of a mammary cell line. Mol Biol Cell.

[29] Chen X, Tukachinsky H, Huang CH, Jao C, Chu YR, Tang HY (2011). Processing and turnover of the Hedgehog protein in the endoplasmic reticulum. J Cell Biol.

[30] Huang CH, Hsiao HT, Chu YR, Ye Y, Chen X (2013). Derlin2 protein facilitates HRD1-mediated retro-translocation of sonic hedgehog at the endoplasmic reticulum. J Biol Chem.

[31] Porter JA, Ekker SC, Park WJ, von Kessler DP, Young KE, Chen CH (1996). Hedgehog patterning activity: role of a lipophilic modification mediated by the carboxy-terminal autoprocessing domain. Cell.

[32] Lee JJ, Ekker SC, von Kessler DP, Porter JA, Sun BI, Beachy PA (1994). Autoproteolysis in hedgehog protein biogenesis. Science.

[33] Porter JA, Young KE, Beachy PA (1996). Cholesterol modification of hedgehog signaling proteins in animal development. Science.

[34] Buglino JA, Resh MD (2008). Hhat is a palmitoylacyltransferase with specificity for N-palmitoylation of Sonic Hedgehog. J Biol Chem.

[35] Chamoun Z, Mann RK, Nellen D, von Kessler DP, Bellotto M, Beachy PA (2001). Skinny hedgehog, an acyltransferase required for palmitoylation and activity of the hedgehog signal. Science.

[36] Pepinsky RB, Zeng C, Wen D, Rayhorn P, Baker DP, Williams KP (1998). Identification of a palmitic acid-modified form of human Sonic hedgehog. J Biol Chem.

[37] Callejo A, Torroja C, Quijada L, Guerrero I (2006). Hedgehog lipid modifications are required for Hedgehog stabilization in the extracellular matrix. Development.

[38] Gallet A, Ruel L, Staccini-Lavenant L, Therond PP (2006). Cholesterol modification is necessary for controlled planar long-range activity of Hedgehog in Drosophila epithelia. Development.

[39] Palm W, Swierczynska MM, Kumari V, Ehrhart-Bornstein M, Bornstein SR, Eaton S (2013). Secretion and signaling activities of lipoprotein-associated hedgehog and non-sterol-modified hedgehog in flies and mammals. PLoS Biol.

[40] Tokhunts R, Singh S, Chu T, D'Angelo G, Baubet V, Goetz JA (2010). The full-length unprocessed hedgehog protein is an active signaling molecule. J Biol Chem.

[41] Amanai K, Jiang J (2001). Distinct roles of Central missing and Dispatched in sending the Hedgehog signal. Development.

[42] Burke R, Nellen D, Bellotto M, Hafen E, Senti KA, Dickson BJ (1999). Dispatched, a novel sterol-sensing domain protein dedicated to the release of cholesterol-modified hedgehog from signaling cells. Cell.

[43] Callejo A, Bilioni A, Mollica E, Gorfinkiel N, Andres G, Ibanez C (2011). Dispatched mediates Hedgehog basolateral release to form the long-range morphogenetic gradient in the Drosophila wing disk epithelium. Proc Natl Acad Sci U S A.

[44] Kawakami T, Kawcak T, Li YJ, Zhang W, Hu Y, Chuang PT (2002). Mouse dispatched mutants fail to distribute hedgehog proteins and are defective in hedgehog signaling. Development.

[45] Ma Y, Erkner A, Gong R, Yao S, Taipale J, Basler K (2002). Hedgehog-mediated patterning of the mammalian embryo requires transporter-like function of dispatched. Cell.

[46] Creanga A, Glenn TD, Mann RK, Saunders AM, Talbot WS, Beachy PA (2012). Scube/You activity mediates release of dually lipid-modified Hedgehog signal in soluble form. Genes Dev.

[47] Hollway GE, Maule J, Gautier P, Evans TM, Keenan DG, Lohs C (2006). Scube2 mediates Hedgehog signalling in the zebrafish embryo. Dev Biol.

[48] Kawakami A, Nojima Y, Toyoda A, Takahoko M, Satoh M, Tanaka H (2005). The zebrafish-secreted matrix protein you/scube2 is implicated in long-range regulation of hedgehog signaling. Curr Biol.

[49] Tsai MT, Cheng CJ, Lin YC, Chen CC, Wu AR, Wu MT (2009). Isolation and characterization of a secreted, cell-surface glycoprotein SCUBE2 from humans. Biochem J.

[50] Woods IG, Talbot WS (2005). The you gene encodes an EGF-CUB protein essential for Hedgehog signaling in zebrafish. PLoS Biol.

[51] Jakobs P, Schulz P, Schurmann S, Niland S, Exner S, Rebollido-Rios R (2017). Ca(2+) coordination controls sonic hedgehog structure and its Scube2-regulated release. J Cell Sci.

[52] Avanesov A, Honeyager SM, Malicki J, Blair SS (2012). The role of glypicans in Wnt inhibitory factor-1 activity and the structural basis of Wif1’s effects on Wnt and Hedgehog signaling. PLoS Genet.

[53] Bilioni A, Sanchez-Hernandez D, Callejo A, Gradilla AC, Ibanez C, Mollica E (2013). Balancing Hedgehog, a retention and release equilibrium given by Dally, Ihog, Boi and shifted/DmWif. Dev Biol.

[54] Glise B, Miller CA, Crozatier M, Halbisen MA, Wise S, Olson DJ (2005). Shifted, the Drosophila ortholog of Wnt inhibitory factor-1, controls the distribution and movement of Hedgehog. Dev Cell.

[55] Gorfinkiel N, Sierra J, Callejo A, Ibanez C, Guerrero I (2005). The Drosophila ortholog of the human Wnt inhibitor factor Shifted controls the diffusion of lipid-modified Hedgehog. Dev Cell.

[56] Lewis PM, Dunn MP, McMahon JA, Logan M, Martin JF, St-Jacques B (2001). Cholesterol modification of sonic hedgehog is required for long-range signaling activity and effective modulation of signaling by Ptc1. Cell.

[57] Gross JC, Chaudhary V, Bartscherer K, Boutros M (2012). Active Wnt proteins are secreted on exosomes. Nat Cell Biol.

[58] Alexandre C, Baena-Lopez A, Vincent JP (2014). Patterning and growth control by membrane-tethered Wingless. Nature.

[59] D'Angelo G, Matusek T, Pizette S, Therond PP (2015). Endocytosis of Hedgehog through dispatched regulates long-range signaling. Dev Cell.

[60] Yamazaki Y, Palmer L, Alexandre C, Kakugawa S, Beckett K, Gaugue I (2016). Godzilla-dependent transcytosis promotes Wingless signalling in Drosophila wing imaginal discs. Nat Cell Biol.

[61] Banziger C, Soldini D, Schutt C, Zipperlen P, Hausmann G, Basler K (2006). Wntless, a conserved membrane protein dedicated to the secretion of Wnt proteins from signaling cells. Cell.

[62] Bartscherer K, Pelte N, Ingelfinger D, Boutros M (2006). Secretion of Wnt ligands requires Evi, a conserved transmembrane protein. Cell.

[63] Goodman RM, Thombre S, Firtina Z, Gray D, Betts D, Roebuck J (2006). Sprinter: a novel transmembrane protein required for Wg secretion and signaling. Development.

[64] Yu J, Chia J, Canning CA, Jones CM, Bard FA, Virshup DM (2014). WLS retrograde transport to the endoplasmic reticulum during Wnt secretion. Dev Cell.

[65] Belenkaya TY, Han C, Yan D, Opoka RJ, Khodoun M, Liu H (2004). Drosophila Dpp morphogen movement is independent of dynamin-mediated endocytosis but regulated by the glypican members of heparan sulfate proteoglycans. Cell.

[66] Franch-Marro X, Marchand O, Piddini E, Ricardo S, Alexandre C, Vincent JP (2005). Glypicans shunt the Wingless signal between local signalling and further transport. Development.

[67] Pan CL, Baum PD, Gu M, Jorgensen EM, Clark SG, Garriga G (2008). C. elegans AP-2 and retromer control Wnt signaling by regulating mig-14/Wntless. Dev Cell.

[68] Port F, Kuster M, Herr P, Furger E, Banziger C, Hausmann G (2008). Wingless secretion promotes and requires retromer-dependent cycling of Wntless. Nat Cell Biol.

[69] Harterink M, Port F, Lorenowicz MJ, McGough IJ, Silhankova M, Betist MC (2011). A SNX3-dependent retromer pathway mediates retrograde transport of the Wnt sorting receptor Wntless and is required for Wnt secretion. Nat Cell Biol.

[70] Pfeiffer S, Ricardo S, Manneville JB, Alexandre C, Vincent JP (2002). Producing cells retain and recycle Wingless in Drosophila embryos. Curr Biol.

[71] Strigini M, Cohen SM (2000). Wingless gradient formation in the Drosophila wing. Curr Biol.

[72] Yamamoto H, Awada C, Hanaki H, Sakane H, Tsujimoto I, Takahashi Y (2013). The apical and basolateral secretion of Wnt11 and Wnt3a in polarized epithelial cells is regulated by different mechanisms. J Cell Sci.

[73] Ayers KL, Gallet A, Staccini-Lavenant L, Therond PP (2010). The long-range activity of Hedgehog is regulated in the apical extracellular space by the glypican Dally and the hydrolase Notum. Dev Cell.

[74] Matusek T, Wendler F, Poles S, Pizette S, D'Angelo G, Furthauer M (2014). The ESCRT machinery regulates the secretion and long-range activity of Hedgehog. Nature.

[75] Tabata T, Kornberg TB (1994). Hedgehog is a signaling protein with a key role in patterning Drosophila imaginal discs. Cell.

[76] Bodeen WJ, Marada S, Truong A, Ogden SK (2017). A fixation method to preserve cultured cell cytonemes facilitates mechanistic interrogation of morphogen transport. Development.

[77] Etheridge LA, Crawford TQ, Zhang S, Roelink H (2010). Evidence for a role of vertebrate Disp1 in long-range Shh signaling. Development.

[78] Ayers KL, Mteirek R, Cervantes A, Lavenant-Staccini L, Therond PP, Gallet A (2012). Dally and Notum regulate the switch between low and high level Hedgehog pathway signalling. Development.

[79] He H, Huang M, Sun S, Wu Y, Lin X (2017). Epithelial heparan sulfate regulates Sonic Hedgehog signaling in lung development. PLoS Genet.

[80] O'Farrell F, Lobert VH, Sneeggen M, Jain A, Katheder NS, Wenzel EM (2017). Class III phosphatidylinositol-3-OH kinase controls epithelial integrity through endosomal LKB1 regulation. Nat Cell Biol.

[81] Lobert VH, Stenmark H (2011). Cell polarity and migration: emerging role for the endosomal sorting machinery. Physiology.

[82] Chabu C, Li DM, Xu T (2017). EGFR/ARF6 regulation of Hh signalling stimulates oncogenic Ras tumour overgrowth. Nat Commun.

[83] Steinhauer J, Liu HH, Miller E, Treisman JE (2013). Trafficking of the EGFR ligand Spitz regulates its signaling activity in polarized tissues. J Cell Sci.

[84] Korkut C, Ataman B, Ramachandran P, Ashley J, Barria R, Gherbesi N (2009). Trans-synaptic transmission of vesicular Wnt signals through Evi/Wntless. Cell.

[85] Beckett K, Monier S, Palmer L, Alexandre C, Green H, Bonneil E (2013). Drosophila S2 cells secrete wingless on exosome-like vesicles but the wingless gradient forms independently of exosomes. Traffic.

[86] Beer KB, Wehman AM (2017). Mechanisms and functions of extracellular vesicle release in vivo-What we can learn from flies and worms. Cell Adhes Migr.

[87] Vyas N, Walvekar A, Tate D, Lakshmanan V, Bansal D, Lo Cicero A (2014). Vertebrate Hedgehog is secreted on two types of extracellular vesicles with different signaling properties. Sci Rep.

[88] Koles K, Nunnari J, Korkut C, Barria R, Brewer C, Li Y (2012). Mechanism of evenness interrupted (Evi)-exosome release at synaptic boutons. J Biol Chem.

[89] Ramirez-Weber FA, Kornberg TB (1999). Cytonemes: cellular processes that project to the principal signaling center in Drosophila imaginal discs. Cell.

[90] Cohen M, Georgiou M, Stevenson NL, Miodownik M, Baum B (2010). Dynamic filopodia transmit intermittent Delta-Notch signaling to drive pattern refinement during lateral inhibition. Dev Cell.

[91] De Joussineau C, Soule J, Martin M, Anguille C, Montcourrier P, Alexandre D (2003). Delta-promoted filopodia mediate long-range lateral inhibition in Drosophila. Nature.

[92] Hamada H, Watanabe M, Lau HE, Nishida T, Hasegawa T, Parichy DM (2014). Involvement of Delta/Notch signaling in zebrafish adult pigment stripe patterning. Development.

[93] Huang H, Kornberg TB (2015). Myoblast cytonemes mediate Wg signaling from the wing imaginal disc and Delta-Notch signaling to the air sac primordium. elife.

[94] Peng Y, Han C, Axelrod JD (2012). Planar polarized protrusions break the symmetry of EGFR signaling during Drosophila bract cell fate induction. Dev Cell.

[95] Roy S, Hsiung F, Kornberg TB (2011). Specificity of Drosophila cytonemes for distinct signaling pathways. Science.

[96] Hsiung F, Ramirez-Weber FA, Iwaki DD, Kornberg TB (2005). Dependence of Drosophila wing imaginal disc cytonemes on Decapentaplegic. Nature.

[97] Roy S, Huang H, Liu S, Kornberg TB (2014). Cytoneme-mediated contact-dependent transport of the Drosophila decapentaplegic signaling protein. Science.

[98] Luz M, Spannl-Muller S, Ozhan G, Kagermeier-Schenk B, Rhinn M, Weidinger G (2014). Dynamic association with donor cell filopodia and lipid-modification are essential features of Wnt8a during patterning of the zebrafish neuroectoderm. PLoS One.

[99] Couto A, Mack NA, Favia L, Georgiou M (2017). An apicobasal gradient of Rac activity determines protrusion form and position. Nat Commun.

[100] Hagemann AI, Kurz J, Kauffeld S, Chen Q, Reeves PM, Weber S (2014). In vivo analysis of formation and endocytosis of the Wnt/beta-catenin signaling complex in zebrafish embryos. J Cell Sci.

[101] Bishop B, Aricescu AR, Harlos K, O'Callaghan CA, Jones EY, Siebold C (2009). Structural insights into hedgehog ligand sequestration by the human hedgehog-interacting protein HHIP. Nat Struct Mol Biol.

[102] Mii Y, Taira M (2011). Secreted Wnt “inhibitors” are not just inhibitors: regulation of extracellular Wnt by secreted Frizzled-related proteins. Dev Growth Differentiation.

[103] Georgiou M, Baum B (2010). Polarity proteins and Rho GTPases cooperate to spatially organise epithelial actin-based protrusions. J Cell Sci.

[104] Ho HY, Rohatgi R, Lebensohn AM, Le M, Li J, Gygi SP (2004). Toca-1 mediates Cdc42-dependent actin nucleation by activating the N-WASP-WIP complex. Cell.

[105] Snyder JC, Rochelle LK, Marion S, Lyerly HK, Barak LS, Caron MG (2015). Lgr4 and Lgr5 drive the formation of long actin-rich cytoneme-like membrane protrusions. J Cell Sci.

[106] Carmon KS, Gong X, Yi J, Wu L, Thomas A, Moore CM (2017). LGR5 receptor promotes cell-cell adhesion in stem cells and colon cancer cells via the IQGAP1-Rac1 pathway. J Biol Chem.

[107] Scopelliti A, Cordero JB, Diao F, Strathdee K, White BH, Sansom OJ (2014). Local control of intestinal stem cell homeostasis by enteroendocrine cells in the adult Drosophila midgut. Curr Biol.

[108] Corbit KC, Aanstad P, Singla V, Norman AR, Stainier DY, Reiter JF (2005). Vertebrate Smoothened functions at the primary cilium. Nature.

[109] Rohatgi R, Milenkovic L, Scott MP (2007). Patched1 regulates hedgehog signaling at the primary cilium. Science.

[110] Haycraft CJ, Banizs B, Aydin-Son Y, Zhang Q, Michaud EJ, Yoder BK (2005). Gli2 and Gli3 localize to cilia and require the intraflagellar transport protein polaris for processing and function. PLoS Genet.

[111] Kornberg TB (2014). The contrasting roles of primary cilia and cytonemes in Hh signaling. Dev Biol.

[112] Yavari A, Nagaraj R, Owusu-Ansah E, Folick A, Ngo K, Hillman T (2010). Role of lipid metabolism in smoothened derepression in hedgehog signaling. Dev Cell.

[113] Jiang K, Liu Y, Fan J, Zhang J, Li XA, Evers BM (2016). PI(4)P Promotes phosphorylation and conformational change of Smoothened through interaction with its C-terminal tail. PLoS Biol.

[114] Manikowski D, Kastl P, Grobe K. Taking the Ocam's Razor approach to Hedgehog lipidation and its role in development. J Dev Biol. 2018;6 10.3390/jdb010003. 10.3390/jdb6010003 PMC5875562 29615552

[115] Cardozo MJ, Sanchez-Arrones L, Sandonis A, Sanchez-Camacho C, Gestri G, Wilson SW (2014). Cdon acts as a Hedgehog decoy receptor during proximal-distal patterning of the optic vesicle. Nat Commun.

[116] Sagar PF, Wiegreffe C, Scaal M (2015). Communication between distant epithelial cells by filopodia-like protrusions during embryonic development. Development.

[117] Huang YL, Niehrs C (2014). Polarized Wnt signaling regulates ectodermal cell fate in Xenopus. Dev Cell.

[118] Huang H, Kornberg TB (2016). Cells must express components of the planar cell polarity system and extracellular matrix to support cytonemes. elife.

[119] Hsia EYC, Zhang Y, Tran HS, Lim A, Chou YH, Lan G (2017). Hedgehog mediated degradation of Ihog adhesion proteins modulates cell segregation in Drosophila wing imaginal discs. Nat Commun.

